# Novel recombinant adeno-associated viruses for Cre activated and inactivated transgene expression in neurons

**DOI:** 10.3389/fncir.2012.00047

**Published:** 2012-07-27

**Authors:** Arpiar Saunders, Caroline A. Johnson, Bernardo L. Sabatini

**Affiliations:** Department of Neurobiology, Harvard Medical School, Howard Hughes Medical InstituteBoston, MA, USA

**Keywords:** optogenetics, viruses, Cre recombinase, circuits, transgenic, tracing

## Abstract

Understanding the organization of the nervous system requires methods for dissecting the contributions of each component cell type to circuit function. One widely used approach combines genetic targeting of Cre recombinase to specific cell populations with infection of recombinant adeno-associated viruses (rAAVs) whose transgene expression is activated by Cre (“Cre-On”). Distinguishing how the Cre-expressing neurons differ functionally from neighboring Cre-negative neurons requires rAAVs that are inactivated by Cre (“Cre-Off”) and can be used in tandem with Cre-On viruses. Here we introduce two rAAV vectors that are inactivated by Cre and carry different fluorophore and optogenetic constructs. We demonstrate single and dual rAAV systems to achieve Cre-On and Cre-Off expression in spatially-intermingled cell populations of the striatum. Using these systems, we uncovered cryptic genomic interactions that occur between multiple Cre-sensitive rAAVs or between Cre-sensitive rAAVs and somatic Cre-conditional alleles and devised methods to avoid these interactions. Our data highlight both important experimental caveats associated with Cre-dependent rAAV use as well as opportunities for the development of improved rAAVs for gene delivery.

## Introduction

Labeling and manipulating genetically-defined neurons *in vivo* is essential for understanding how specific cell types contribute to brain function, but requires methods to accurately introduce and potently express transgenes. One such method combines transgenic animals in which site-specific recombinases (SSRs) expressed in specific cell populations trigger transgene expression by activating DNA-based recombinant adeno-associated virus (rAAV) vectors (Kuhlman and Huang, 2008; Sohal et al., 2009; Betley and Sternson, 2011).

Cre recombinase, like all SSRs, excises or recombines DNA depending on the relative orientation of short, directional DNA sequences (Tronche et al., 2002). The 34 base pair (bp) lox sites, recognized by Cre, consist of two 13 bp palindromic regions and an intervening non-palindromic 8 bp spacer that determines the orientation of the site. When two lox sites are oriented in the same direction, Cre excises the DNA flanked by the lox sites, leaving a single lox site behind. Conversely, when the lox sites are oriented in the opposite direction, Cre flips the flanked DNA into the antisense orientation. Both reactions involve the exchange of DNA between the two lox sites and are normally reversible (Van Duyne, 2001).

Differences in palindromic or spacer regions of lox sites, either naturally occurring (Siegel et al., 2001) or randomly mutated (Sheren et al., 2007), can confer specificity to Cre recognition. Exploiting lox variants that undergo variant-specific recombination has enabled strategies for making Cre recombination effectively irreversible (Albert et al., 1995; Araki et al., 1997; Atasoy et al., 2008). The FLEx system, first used as a Cre-reporter (Schnütgen et al., 2003) and then applied to rAAV transgenes (Atasoy et al., 2008; Sohal et al., 2009), uses recombination between two pairs of like loxP and lox2272 sites to confer a permanent recombination event. Expression in the presence of Cre (“Cre-On”) is achieved by FLEx recombination of a transgene that changes the orientation of the coding sequence with respect to the promoter from the anti-sense to sense. Conversely, inactivation of expression in the presence of Cre (“Cre-Off”) can be achieved by simply starting the transgene in the sense orientation.

Cre-On rAAVs have been used widely to study the function of Cre-expressing neuron populations (Cardin et al., 2009; Kravitz et al., 2010; Cohen et al., 2012), whereas Cre-Off rAAVs, despite their experimental value, have received only minor attention (Atasoy et al., 2008; Kim et al., 2011). In brain regions of transgenic Cre animals with intermingled cell types, Cre-Off rAAVs can be used to compare morphology, physiological properties, or behavioral function with that of Cre-expressing populations. Whereas transgenic Cre reporter animals that differentially label cells with and without Cre expression are of great utility (Lobe et al., 1999; Novak et al., 2000; Muzumdar et al., 2007), these reporters are of limited use for comparing cell populations from defined brain regions at specific stages of development, since Cre reporting is permanent and occurs throughout the whole animal. Co-infection of Cre-On and Cre-Off rAAVs would allow such comparisons to be made in the same experiment. However, due to our incomplete understanding of the AAV life cycle in cells, it is unclear how transduction of multiple rAAVs with engineered genetic elements may interact with each other and with engineered sites in the host cell genome.

Here we show that the presence of the same lox site variants in the Cre-On and Cre-Off rAAVs prevents their use to simultaneously target two cell populations. To circumvent this problem, new rAAVs were developed for the simultaneous manipulation of multiple cell-populations via Cre-On and Cre-Off expression of transgenes. Using the FLEx system and a third alternative lox site, we created a panel of Cre-Off rAAVs carrying genetically-encoded fluorophores or optogenetic constructs (Table 1) and validated two systems for simultaneous Cre-On and Cre-Off expression. In addition, we identify important caveats of using Cre sensitive rAAVs with genomic Cre conditional alleles and demonstrate how these can be avoided with the new tools.

**Table 1:** **rAAV vectors and transgenes**.

**DIO**	**DO**	**FAS**	**Cre-Switch (DO-DIO)**
mCherry (37083)	mCherry (37119)	TdTomato (37092)	TdTomato-eGFP (37120)
eGFP (37084)	eGFP (37085)	eGFP (37091)	
ChETA-TdTomato (37755)	ChR2(H134R)-mCherry (37082)	ChR2(H134R)-mCherry (37090)	
	ChETA-eYFP (37086)	ChETA-TdTomato (37089)	
	ChETA-TdTomato (37756)	NpHR3.0-eYFP (37088)	
	NpHR3.0-eYFP (37087)		

Addgene accession numbers are listed for each vector.

## Methods

### rAAV cloning and production

Cloning of Double-floxed Inverted Orientation (DIO) Cre-On and Double-floxed Orientation (DO)/FAS Cre-Off vectors was based on the pAAV-Ef1á-DIO-hChR2(H134R)-mCherry-WPRE-pA vector from the Deisseroth Lab (http://www.stanford.edu/group/dlab/optogenetics/sequence_info.html). Cloning and sequence confirmation was done either in house or outsourced (Genscript). DIO and DO vectors were cloned using the Asc1 and Nhe1 restriction sites, introduced at the ends of transgenes through transgene-specific primers and PCR amplification. All transgenes were amplified from existing vectors except for ChETA-TdTomato, which was custom synthesized (Genscript). For DIO cloning, the Asc1 site was N-Terminal and the Nhe1 site C-Terminal with respect to the transgene; for DO cloning, the sites were reversed. All N-Terminal sites included a Kozak sequence (GCCACC) directly preceding the start codon. To generate FAS vectors, a custom insert was synthesized to replace the FLEx insert and sub-cloned into the original pAAV-Ef1α-DIO-hChR2(H134R)-mCherry-WPRE-pA backbone using BamHI and EcoRI sites. The FAS insert preserves the original multiple cloning site of the FLEx insert and contains Asc1 and Nhe1 sites within the FAS sites which were used to subclone the other FAS transgenes. Full maps and clones are available on Addgene (http://www.addgene.org/).

All vectors were amplified with recombination deficient bacteria (OneShot Stbl3, Invitrogen) and tested functionally for Cre-On and Cre-Off expression by calcium phosphate transfection (Invitrogen) into HEK 293 cells, HEK 293 cells constitutively expressing Cre (Langer et al., 2002), or HEK 293 cells co-transfected with fluorophore tagged Cre. After 2–6 days of expression, fluorescence was compared across conditions to ensure that transgene expression was Cre sensitive. Functional vectors were packaged as serotype eight by a commercial vector core facility (University of North Carolina).

### Stereotaxic injections

For intracranial injections, post-natal day 14–40 mice were anesthetized with isofluorane and placed into a stereotaxic apparatus. For co-injections, viruses were mixed at ratios that were adjusted empirically to achieve approximately equal expression levels of GFP and mCherry/TdTomato fluorophores. Virus was delivered at 100nl/min using a Microinject system (WPI). Striatum was targeted by directing the needle approximately 0.9mm anterior and 2.4mm lateral from Bregma and 2.1mm from the pial surface. Cortical injections were targeted using the same anterior–posterior and medial-lateral coordinates, but the needle was directed 0.4mm from the pial surface. Coordinates were adjusted slightly by age and 300–1000nl were injected bilaterally. Following injections and wound closure, mice received ketoprofen (10 mg/kg) for analgesia and were returned to home cages for 3–4 weeks. All surgical procedures, animal handling, and euthanasia were carried out in accordance with federal guidelines and were approved by the Harvard Medical School Institutional Animal Care and Use Committee.

### Fixed tissue preparation, immunostaining, and imaging

Isoflorane anesthetized mice were transcardially perfused with 4% paraformaldehyde and the brains were post-fixed for 1–7 days. Brains were sectioned coronally or sagitally at 40–50 μm thickness using a Vibratome. No immuno-enhancement was used to increase the signal of native fluorophores. For Cre immunohistochemistry, slices were incubated overnight at 4°C with mouse anti-Cre antibody (1:250, Millipore) in 1x PBS containing 5% normal goat serum (GIBCO) and 0.3% TritonX-100. The following day, tissue was rinsed in PBS, reacted with goat anti-mouse Alexa 647 (1:500, Molecular Probes) for 1 h at room temperature in the same blocking buffer as for the primary antibody reaction. Slices were rinsed, mounted onto superfrost slides, dried and coverslipped under ProLong antifade reagent with DAPI (Molecular Probes). Whole-slide images were acquired with a VS110 slice scanning microscope (Olympus). To determine fluorophore co-expression, confocal stacks were taken with a Zeiss LSM 150 or Olympus FV1000 and analyzed using ImageJ.

## Results

**Figure 1:**
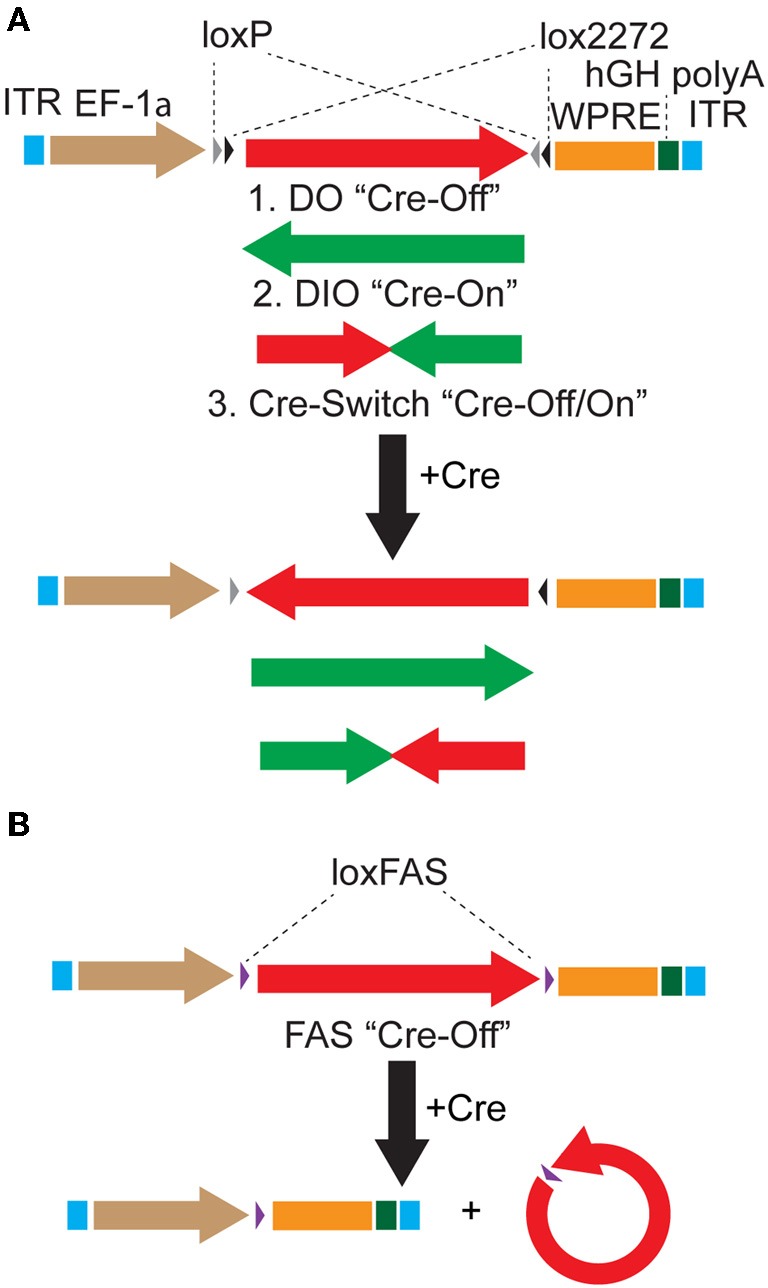
**Strategies to achieve Cre-dependent rAAV transgene expression**. **(A)** Oppositely oriented loxP (gray triangle) and lox2272 (black triangle) sites permit Cre-mediated recombination and inversion of the flanked transgene with respect to the EF-1α promoter. Downstream sequences stabilize the mRNA (woodchuck polyresponse element, WPRE) and trigger polyadenylation (human growth hormone polyadenylation, hGH polyA). After recombination, the transgene is flanked by one loxP and one lox2272 site, which do not recombine efficiently, effectively locking the transgene into position. The starting orientation of the transgene determines the Cre dependence of expression. The double-floxed orientation (1, DO) configuration, in which the open reading frame (ORF) of the transgene begins in the functional orientation with respect to the promoter, maintains expression only in cells lacking Cre (Cre-Off). In the opposite starting orientation, the double-floxed inverted (2, DIO) ORF must be recombined to be functional and expression is achieved only in Cre expressing cells (Cre-On). A single transgene containing two ORFs oriented oppositely with respect to each other and separated by stop codons (3, Cre-Switch) switches expression between the two ORFs depending on Cre expression. For Cre-Switch transgenes, the first, forward orientated ORF is expressed in Cre negative cells whereas the second, inverted ORF is activated in Cre positive cells. ITR = inverted terminal repeats. **(B)** Cre-Off control of transgene expression can also be achieved by Cre-based excision of the ORF using alternative lox FAS sites. loxFAS sites flank the ORF and are oriented in the same direction such that the flanked sequence is excised by Cre.

We used the DIO Cre-On vector pAAV-Ef1α-DIO-hChR2(H134R)-mCherry-WPRE-pA (Sohal et al., 2009) as the parent vector to generate DO Cre-Off vectors by cloning fluorophores and optogenetic constructs in the forward orientation with respect to the Ef1α promoter (Figure 1). These DO vectors (Table 1) express the encoded protein until Cre-mediated recombination reverses the orientation of the transgene, leading to the transcription of non-functional mRNA (Figure 1A). In addition, we generated Cre-Switch vectors in which the coding sequences for two proteins are inserted with inverted orientations relative to each other such that Cre-mediated recombination and inversion switches which protein is expressed (Figure 1A). Lastly, the alternative lox site “FAS” (Siegel et al., 2001) was used to generate an independent FAS Cre-Off vector in which the coding sequence of the protein of interest is flanked by two FAS sites and is excised by Cre recombinase, turning off expression (Figure 1B). All vectors generated for this study, as well as additional Cre-On and Cre-Off viruses of similar design (Table 1), are available from Addgene (http://www.addgene.org/).

To test the Cre-Off vectors, we took advantage of the anatomy of the striatum. The vast majority (>95%) of striatal neurons are medium spiny neurons (MSNs), which provide the only striatal output. MSNs come in two types which can be grouped by axonal projection pattern and dopamine receptor expression (Smith et al., 1998). Indirect pathway (iMSNs) express the dopamine receptor 2 (D2) and project exclusively to the Globus Pallidus (GP). Direct pathway MSNs (dMSNs) express the dopamine receptor 1 (D1) and project primarily to the Substantia Nigra reticulata (SNr) as well as a more minimal axon collateral in the GP (Kawaguchi et al., 1990). Since dMSNs and iMSNs are equal in number, spatially intermingled, and selectively targetable for Cre expression using BAC transgenes (Gong et al., 2003), the anatomy of the striatum provides an ideal test system for Cre-On and Cre-Off rAAVs.

**Figure 2:**
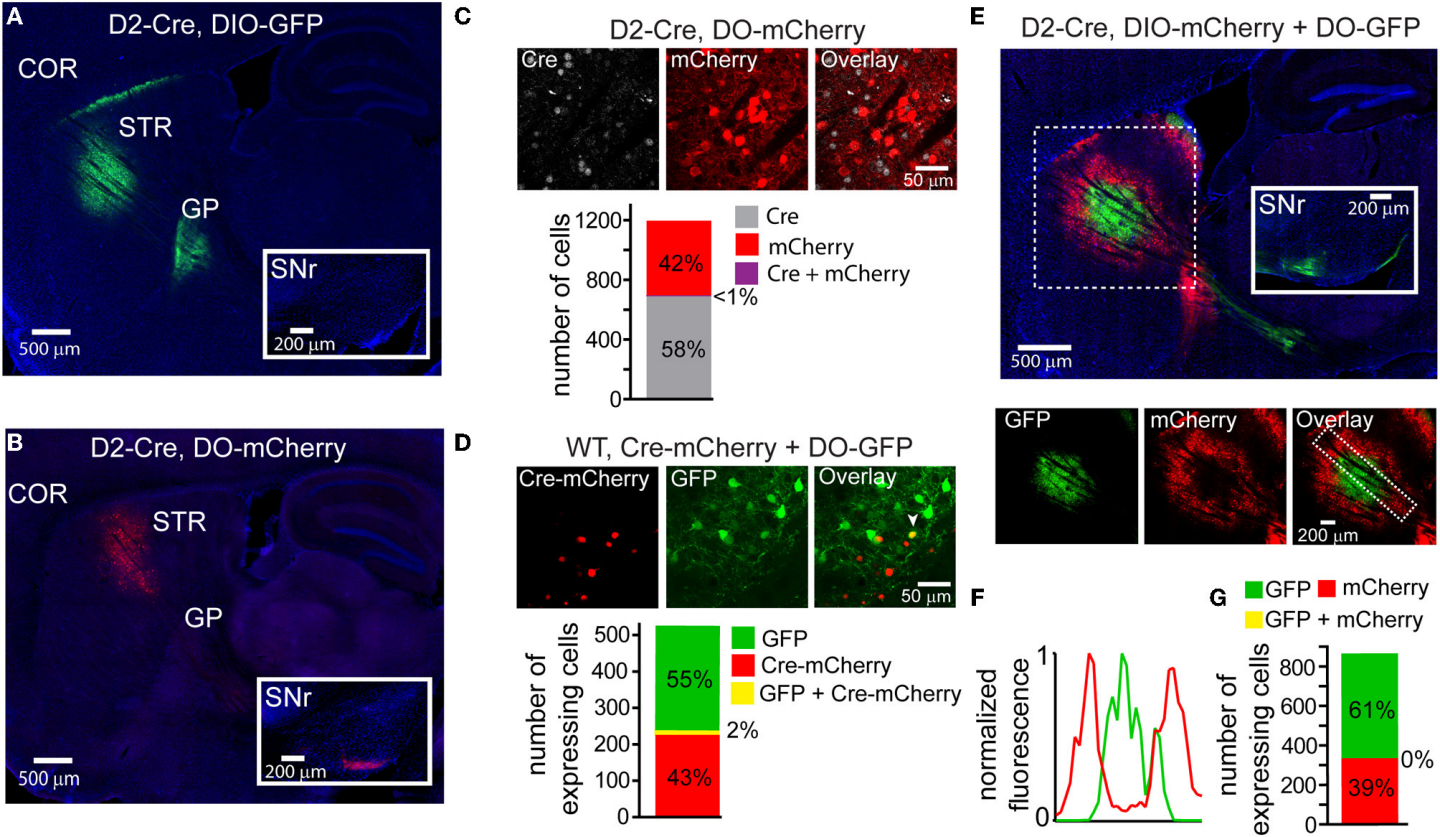
**DIO and DO rAAVs achieve Cre-On and Cre-Off expression but exhibit interfering interactions when co-infected**. **(A)** Sagittal section through a D2-Cre mouse infected with DIO-GFP in the striatum. GFP^+^ axons emanating from the striatal infection innervate the globus pallidus (GP), the target of indirect pathway medium spiny neurons (iMSNs). No GFP^+^ expression is observed in the substantia nigra reticulata (SNr, inset). COR = cortex, STR = striatum. **(B)** Sagittal section through a D2-Cre mouse infected with DO-mCherry in the striatum such that mCherry expression is excluded from iMSNs. mCherry^+^ axons leaving the striatum innervate the SNr (inset), consistent with expression in direct pathway MSNs (dMSNs). **(C)** Antibody staining against Cre in D2-Cre striatum infected with DO-mCherry reveals that Cre efficiently turns off mCherry expression, indicative of Cre-Off behavior. *Top*, a single confocal plane showing mCherry expression excluded from cells with Cre^+^ nuclei. *Bottom,* quantification of co-expression of Cre and mCherry (*N* = 4 infections, *N* = 1187 cells) reveals that mCherry expression (*N* = 495 cells) was almost completely excluded from Cre^+^ cells (*N* = 1/691 cells double-positive for mCherry and Cre). **(D)** Co-infection of rAAVs encoding a Cre-mCherry fusion and DO-GFP into the striatum of a wild type (WT) mouse efficiently prevents GFP expression in Cre-mCherry expressing cells. *Top,* a single confocal plane containing interspersed mCherry^+^ and GFP^+^ cells. Note that the mCherry expression is nuclear due to nuclear targeting of Cre. The arrowhead indicates a cell co-expressing mCherry and GFP. *Bottom*, confocal imaging of infected tissue (*N* = 2 infections, *N* = 521 cells) reveals that cells expressing Cre-mCherry (*N* = 237) and cells expressing GFP (*N* = 284) are largely non-overlapping (*N* = 11/521 cells were double-positive for both fluorophores). **(E)** Sagittal section through a D2-Cre mouse infected with DIO-mCherry and DO-GFP in the striatum. Despite the physical intermixing of Cre positive and negative neurons in the striatum, the resulting GFP and mCherry expression is spatially segregated. *Below*, separate visualization of the green and red fluorescence channels demonstrates that mCherry is excluded from the striatal volume expressing GFP. **(F)** Normalized mean fluorescent values for GFP and mCherry quantified from boxed inset in **E**, *bottom right*. **(G)** Confocal imaging around the border regions (*N* = 2 infections, *N* = 865 cells) containing both GFP^+^ (*N* = 529) and mCherry^+^ (*N* = 336) cells revealed no overlap in expression.

Using transgenic mice in which Cre expression is driven by the dopamine receptor 2 regulatory sequences (D2-Cre) (Gong et al., 2003) and thus restricted to iMSNs, we infected the striata with DIO-GFP or DO-mCherry rAAVs and compared the GFP/mCherry^+^ projections in sagittal slices of fixed tissue 3–4 weeks post-infection. As expected, DIO-GFP expression was restricted to projections to the GP, the sole innervation zone of iMSNs (Figure 2A). Conversely, DO-mCherry expression was restricted to projections that provided a very minimal arbor in the GP but a large arbor in the SNr, the main innervation zone of dMSNs (Figure 2B). These anatomical results suggest that DO-GFP expression was excluded from Cre-expressing iMSNs.

To quantify the efficiency with which stable, transgenic Cre expression turns off DO rAAV expression, Cre-expressing (Cre^+^) neurons in the striatum of D2-Cre mice infected with DO-GFP were identified with an antibody against Cre. Confocal imaging of the infection zone and quantification of the number of cells positive for Cre and/or GFP revealed that of 692 Cre^+^ and 496 GFP^+^ cells (*N* = 4 infections) only one cell was double-positive (Figure 2C). Similarly, to quantify the ability of virally introduced Cre to turn off DO expression, we infected the striata of wild-type mice simultaneously with rAAVs carrying Cre-mCherry and DO-GFP at titers that resulted in roughly equal numbers of cells expressing Cre-mCherry and GFP (Figure 2D). After 3–4 weeks of infection, of 295 Cre-mCherry^+^ and 237 GFP^+^ cells, 11 cells were double positive (*N* = 4 infections). Thus, either constitutive expression of Cre from a BAC or simultaneous viral introduction of Cre efficiently turns off expression of DO rAAVs.

## Interference of expression with co-infection of DIO and DO viruses

Many experiments would benefit from simultaneous expression of different transgenes in Cre-expressing and non-Cre expressing cell populations. We tested whether DIO and DO rAAVs can be used to target expression to distinct cell populations by simultaneously infecting the striata of D2-Cre mice with DIO-mCherry and DO-GFP (Figure 2E). While both mCherry^+^ and GFP^+^ cells were observed in striatum, their fluorescent signals were not intermingled: GFP expression dominated around the injection site, whereas mCherry expression was strongest on the edges of the infection volume. This mutually exclusive expression pattern was quantified by comparing the GFP and mCherry fluorescence signals in the infected area (Figure 2F). Despite the lack of intermingled expression, the Cre dependence of the Cre-On and Cre-Off rAAVs was maintained, as GFP^+^ and mCherry^+^ axons were restricted to the GP and SNr, respectively, and confocal imaging in the small border areas containing intermingled GFP and mCherry expressing cells demonstrated non-overlapping expression at the cellular level: of 336 mCherry^+^ cells and 529 GFP^+^ cells, none expressed both fluorophores (*N* = 4 infections) (Figure 2G).

## Cre-Switch and alternative Cre-Off systems

**Figure 3:**
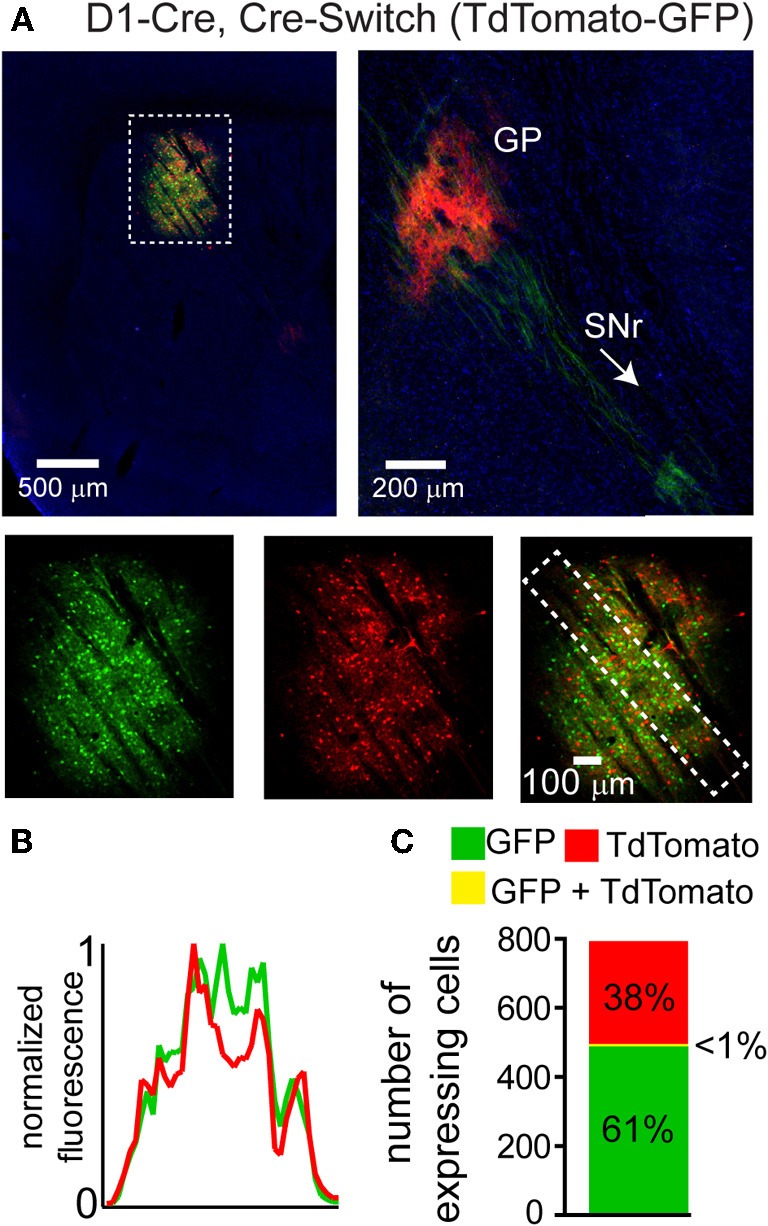
**A single orientation switching transgene can mediate Cre-On and Cre-Off expression without spatial interference**. **(A)** *Left*, Sagittal section through a D1-Cre mouse infected with a Cre-Switch transgene encoding Cre-Off TdTomato and Cre-On GFP in the striatum. *Right*, more medial section containing the GP and both TdTomato^+^ and GFP^+^ projections. *Bottom*, GFP and TdTomato fluorescence is spatially intermingled. **(B)** Normalized mean fluorescent values for GFP and TdTomato quantified from boxed inset in A, *bottom right*. **(C)** Confocal imaging through the infection volume (*N* = 3 infections, *N* = 796 cells) revealed that GFP (*N* = 489 cells) and TdTomato (*N* = 302 cells) were expressed in largely non-overlapping cell populations (*N* = 7/791 cells were double-positive for GFP and TdTomato).

We hypothesized that the lack of DIO Cre-On expression in striatal volumes with strong DO Cre-Off expression was due to the presence of loxP and lox2272 sites in both vectors. We therefore designed two new Cre-sensitive rAAV vectors to achieve simultaneous Cre-On and Cre-Off expression. We tested whether differential Cre-On and Cre-Off expression could be achieved using Cre-mediated inversion of a single transgene (Schnütgen et al., 2003). This “Cre-Switch” transgene encodes two open reading frames positioned back-to-back but in inverted orientations such that the coding sequence located next to the promoter is in the sense orientation (Figure 1A). We synthesized a Cre-Switch transgene encoding Cre-Off TdTomato and Cre-On GFP, and injected the rAAV containing this construct into the striata of mice where Cre is expressed behind the dopamine receptor 1 regulatory sequences (D1-Cre) and is thus restricted to dMSNs. (Figure 3) (Gong et al., 2003). GFP and TdTomato expressing cells were intermingled within the striatum (Figure 3A, top left) and quantification of normalized average GFP and TdTomato signals revealed no spatial interference (Figure 3B). Consistent with differential Cre expression, Cre-On GFP^+^ projections were observed in the SNr, while Cre-Off TdTomato^+^ projections were visible in the GP (Figure 3A, top right). Confocal microscopy revealed that of 494 GFP^+^ cells, and 307 TdTomato^+^ cells, only five cells were double-positive (*N* = 3 infections) (Figure 3C). Since TdTomato is expressed in the absence of Cre, the larger proportion of GFP^+^ vs. TdTomato^+^ cells likely reflects undetectably low levels of TdTomato expression rather than inefficient recombination. Thus Cre-Switch transgenes efficiently target the expression of different transgenes to Cre-expressing and non-expressing cell populations.

**Figure 4:**
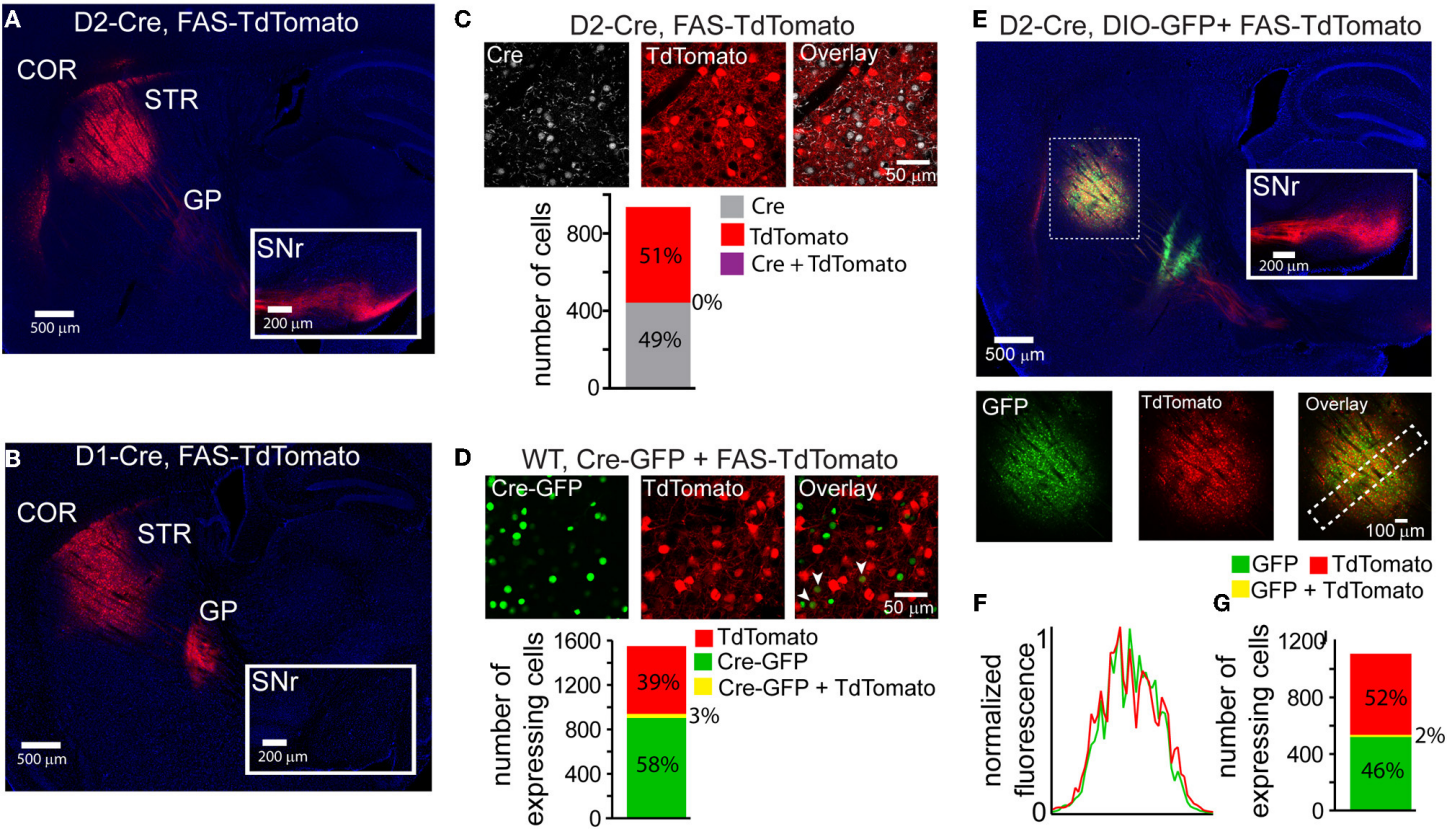
**LoxFAS excision achieves efficient Cre-Off rAAV expression that does not interfere with DIO Cre-On expression**. **(A)** Sagittal section through a D2-Cre mouse infected with FAS-TdTomato in the striatum. TdTomato is excluded from iMSNs. TdTomato^+^ axons arborize in the SNr (inset) but only minimally in the GP, consistent with expression in dMSNs. **(B)** Sagittal section through a D1-Cre mouse infected with FAS-TdTomato in the striatum. TdTomato expression is excluded from dMSNs. TdTomato^+^ axons leaving the striatum target the GP, but not the SNr (inset), consistent with exclusion from dMSNs. **(C)** Antibody staining against Cre in D2-Cre striatum infected with FAS-TdTomato reveals efficient Cre-Off expression in the presence of Cre. *Top*, a single confocal plane showing Cre^+^ nuclei excluded from interspersed TdTomato^+^ cells. *Bottom*, confocal imaging through infected and stained tissue volumes (*N* = 4 infections, *N* = 929 cells) revealed that TdTomato expression (*N* = 477 cells) was completely absent in Cre^+^ cells (*N* = 452). **(D)** Co-infection of rAAVs Cre-GFP and FAS-TdTomato into the striatum of a wild type (WT) mouse leads to efficient exclusion of TdTomato in Cre-GFP expressing cells. *Top,* a single confocal plane containing interspersed GFP^+^ and TdTomato^+^ cells. Note that the GFP expression is nuclear due to the targeting of Cre. The arrowheads indicate cells co-expressing GFP and TdTomato. *Bottom*, confocal imaging of volumes of infected tissue (four infections, *N* = 1258 cells) reveal that a small fraction of the total GFP expressing cells (*N* = 948) also expressed TdTomato (*N* = 40 cells). Conversely, the majority of TdTomato^+^ cells (*N* = 610) did not express Cre-GFP. **(E)** Sagittal section through a D2-Cre mouse infected with DIO-GFP and FAS-TdTomato in the striatum. GFP^+^ axons arborize in the GP, whereas TdTomato^+^ axons pass through GP en route to the SNr. *Bottom*, GFP, and TdTomato signals exhibit spatially intermingled expression patterns. **(F)** Normalized mean fluorescent values for GFP and TdTomato quantified from boxed inset in **E**, *bottom right*. GFP and TdTomato expression levels mirror each other throughout the infection area. **(G)** Confocal imaging through infected volumes (*N* = 3 infections, *N* = 1116 cells) revealed that DIO and FAS co-infection targeted expression to mostly non-overlapping cell populations (FAS-TdTomato, *N* = 578 cells; DIO-GFP, *N* = 519; FAS-TdTomato and DIO-GFP, *N* = 19).

Cre-Switch transgenes are limited both by the small packaging capacity of rAAVs and the cost associated with building unique rAAVs to achieve the desired combinations of transgenes for expression targeting. We therefore designed an alternative Cre-Off rAAV vector that could be co-infected with DIO Cre-On vectors without spatial interference. Based on our hypothesis that spatial interference results from shared lox sites on the Cre-On and Cre-Off vectors, we designed a set of Cre-Off rAAVs that uses the FAS lox site variant (Table 1). LoxFAS sites do not undergo efficient recombination with either loxP or lox2272 in bacteria (Siegel et al., 2001). To achieve Cre-Off expression, the sense oriented rAAV transgene was flanked by loxFAS sites oriented in the same direction (Figure 1B), leading to Cre-mediated excision of the flanked transgene. The Cre-Off FAS vector, henceforth called FAS, was tested by injecting FAS-tdTomato into the striata of D2-Cre and D1-Cre mice and examining the labeled projections (Figure 4). Cre expression in iMSNs resulted in large TdTomato^+^ arborizations to the SNr but only minor arborizations in GP (Figure 4A) whereas Cre expression in dMSNs resulted in a large arborization in GP and no expression in SNr (Figure 4B), indicating that Cre efficiently eliminates FAS rAAV transgene expression.

The efficiency of stable transgenic Cre expression in turning off FAS expression was quantified in fixed slices of striatum of D2-Cre mice infected with FAS-TdTomato and immunostained for Cre (Figure 4C). Of 477 TdTomato^+^ cells and 452 Cre^+^ cells, no cells were double-positive (*N* = 4 infections). Thus stable transgenic Cre expression efficiently eliminates FAS rAAV transgene expression, leading to undetectable fluorophore expression within 3–4 weeks.

To quantify the efficiency of virally introduced Cre to turn off FAS expression, we infected the striata of wild-type mice simultaneously with rAAVs carrying Cre-GFP and FAS-TdTomato at titers where approximately equal numbers of cells expressed Cre-mCherry and GFP (Figure 4D). We used confocal microscopy to image areas of the infection zone with interspersed Cre-GFP and TdTomato expressing cells and quantified the number of cells positive for Cre-GFP, TdTomato, or double-positive for both. Of 948 Cre-GFP^+^ and 650 TdTomato^+^ cells, 40 were double-positive (*N* = 4 infections). Thus viral introduction of Cre efficiently turns off expression of simultaneously infected FAS rAAVs.

To test whether Cre-Off FAS and Cre-On DIO rAAVs could be co-infected without spatial interference, FAS-TdTomato and DIO-GFP were injected into the striatum of D2-Cre mice (Figure 4E). GFP^+^ and TdTomato^+^ cells were intermingled in the striatum and did not exhibit interference (Figure 4F). GFP^+^ but not TdTomato^+^ projections were observed in the GP and only TdTomato^+^ were observed en route to the SNr, consistent with Cre-On GFP expression in iMSNs and Cre-Off TdTomato expression in dMSNs. Confocal microscopy revealed that of 519 GFP^+^ cells, and 578 TdTomato^+^ cells, 19 cells were double-positive (Figure 4G) (*N* = 4 infections). Thus co-infection of FAS and DIO rAAVs targets efficient Cre-Off and Cre-On expression without spatial interference.

## Cre-On/Off viral use in mice with Cre-conditional alleles

**Figure 5:**
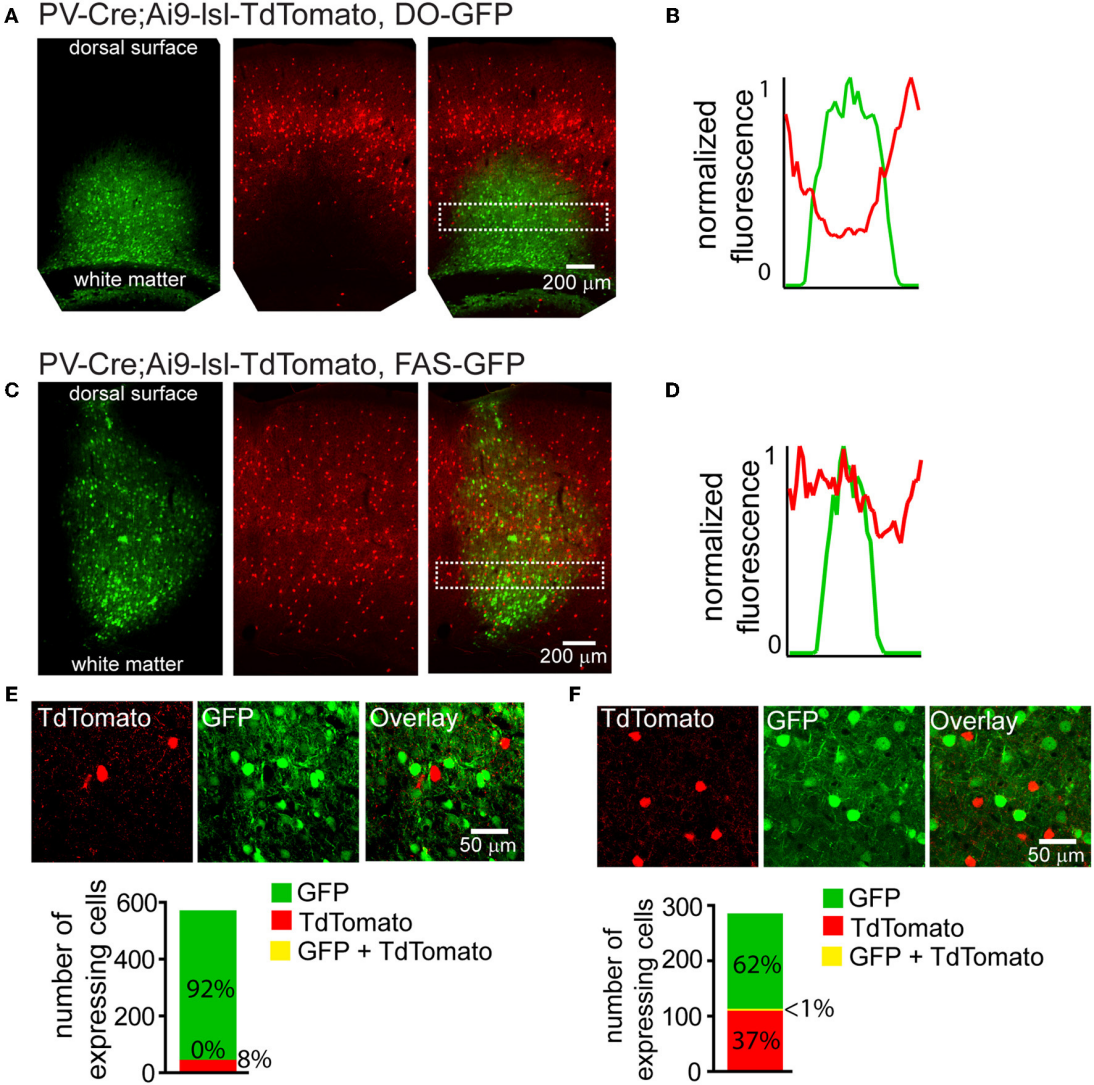
**Cre-Off infection with DO rAAV interferes with the expression of fluorescent Cre reporter allele whereas infection with FAS rAAV does not**. **(A)** Coronal section through the cortex of PV-Cre; Ai9-lsl-TdTomato mouse after infection with DO-GFP. Separate GFP and TdTomato fluorescent channels demonstrate that Cre reporter fluorescence is drastically reduced in the area of DO-GFP infection. **(B)** Normalized mean fluorescent values for GFP and TdTomato quantified from boxed inset in **A**. **(C)** Coronal section through the cortex of PV-Cre; Ai9-lsl-TdTomato mouse after infection with FAS-GFP. Separate GFP and TdTomato fluorescent channels demonstrate that Cre reporter fluorescence is not reduced in the area of FAS-GFP infection. **(D)** Normalized mean fluorescent values for GFP and TdTomato quantified from boxed inset in **C**. **(E)** DO-GFP expression is excluded from remaining TdTomato^+^ cells in the conditions shown in **(A)**. *Top,* a single confocal plane at the border region of the infection containing both TdTomato^+^ and GFP^+^ cells. *Bottom*, confocal imaging of volumes of infected tissue (*N* = 2 infections, *N* = 527 GFP^+^ cells, *N* = 43 TdTomato^+^ cells) reveal GFP expression is excluded from TdTomato^+^ cells. **(F)** FAS-GFP expression is excluded from TdTomato^+^ cells in the conditions shown in **C**. *Top,* a single confocal plane of the infection containing both TdTomato^+^ and GFP^+^ cells. *Bottom*, confocal imaging of volumes of infected tissue (*N* = 2 infections, *N* = 177 GFP^+^ cells, *N* = 105 TdTomato^+^ cells) reveal GFP expression is almost completely excluded from TdTomato^+^ cells (*N* = 1/282 cells double-positive for GFP and TdTomato).

Cre-reporter alleles are used to visualize the spatial distribution of cells that have expressed Cre at any point in development. Cre activates the reporter by excising a stop cassette flanked by loxP sites and located between the promoter and the reporter gene (Madisen et al., 2009). Many anatomical or physiological experiments would benefit from simultaneous labeling of a Cre-reporter and Cre-On or Cre-Off rAAVs. We used a mouse in which Cre expression is driven under the regulatory control of Parvalbumin and reported by TdTomato fluorescence (PV-Cre;Ai9-lsl-tdTomato) to test whether Cre-Off rAAV labeling could be used in conjunction with a Cre reporter allele (Madisen et al., 2009). Injection of DO-GFP into the cortex of PV-Cre; Ai9-lsl-tdTomato mouse, resulted in a robust drop in TdTomato reporter fluorescence in the infection area (Figure 5A), indicating shut-down of the previously active genomic allele. Quantification of normalized average GFP and TdTomato signals illustrates this inverse relationship between expression levels of the two fluorophores (Figure 5B). In contrast, when Cre-Off FAS-GFP was injected, no reduction in TdTomato reporter fluorescence was observed (Figure 5C). Quantification of normalized average GFP and TdTomato signals illustrates no spatial interference between the two fluorophores in this case (Figure 5D).

Confocal microscopy demonstrated that fluorophore expression from DO- and FAS-based Cre-off viruses was efficiently excluded from PV-Cre cortical interneurons. Of 527 DO-GFP^+^ cells and 43 TdTomato^+^ cells, none were double positive (*N* = 2 infections) (Figure 5E). Of 177 FAS-GFP^+^ cells and 105 TdTomato^+^ cells, 1 was double-positive (*N* = 2 infections) (Figure 5F). Exclusion of GFP from TdTomato^+^ cells suggests that Cre was present in these cortical PV-Cre cells at the time of infection and efficiently recombines or excises the transgene to turn off GFP expression.

## Co-expression of multiple rAAV transgenes

**Figure 6:**
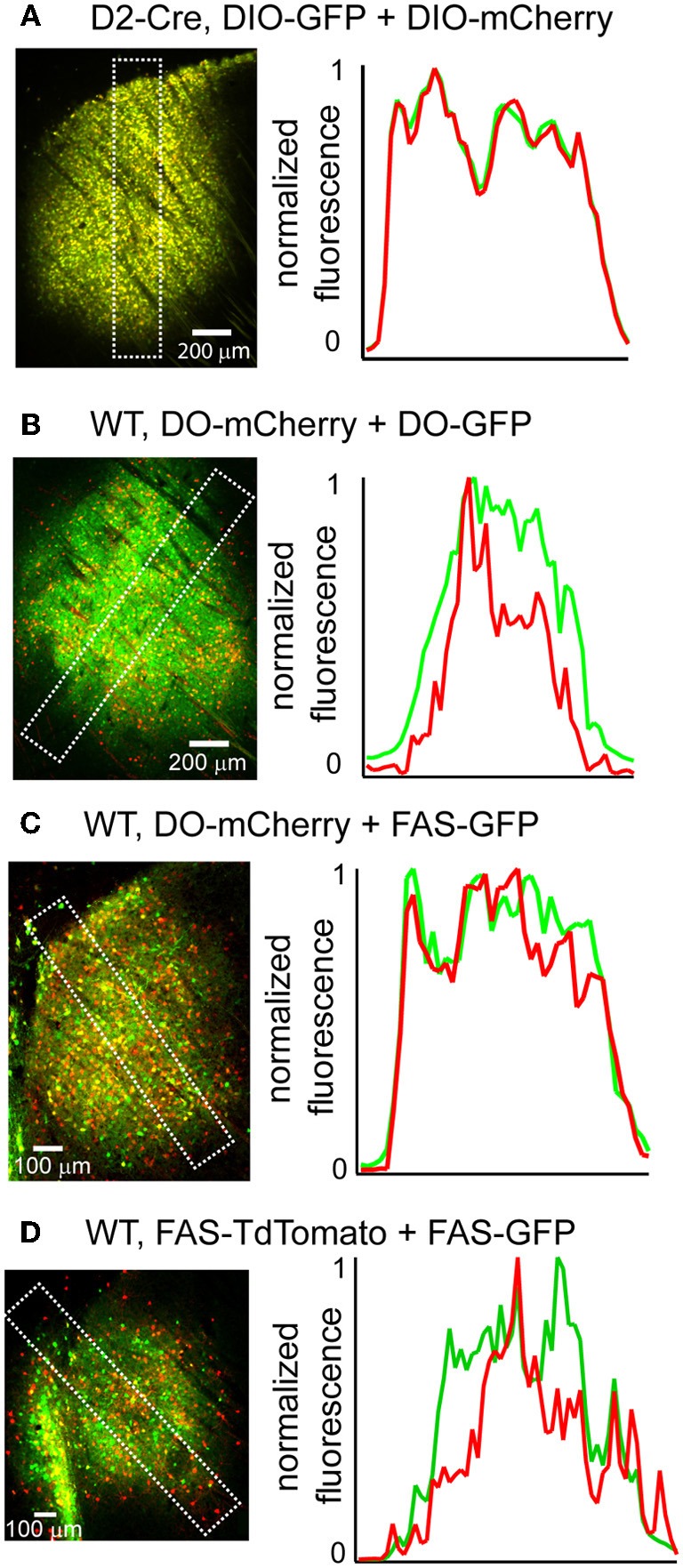
**Co-infection with DIO or DO/FAS rAAVs drives co-expression without spatial interference**. **(A)** Infection of multiple DIO Cre-On rAAVs results in co-expression of transgenes without interference. *Left*, a sagittal section of a D2-Cre mouse infected with DIO-GFP and DIO-mCherry in the striatum. *Right*, normalized mean fluorescent values for GFP and mCherry quantified from boxed inset in **A**. **(B–D)** Infection of multiple Cre-Off rAAVs results in co-expression of transgenes without spatial interference. *Left*, sagittal sections from wild type mice infected with **B**. DO-mCherry + DO-GFP, **C**. DO-mCherry + FAS-GFP, or **D**. FAS-TdTomato + FAS-GFP in the striatum. *Right*, normalized mean fluorescent values for GFP and mCherry/TdTomato quantified from boxed inset in to the left.

Due to the limited packaging capacity of rAAVs, infecting multiple Cre-On or Cre-Off rAAVs may be an effective way to introduce more than one transgene in the same cell populations. We tested whether multiple Cre-On or Cre-Off rAAVs could be co-infected without spatial interference of transgene expression by infecting the striatum and quantifying GFP and mCherry/TdTomato fluorescence. Co-infection of the striatum of D2-Cre mice with DIO-mCherry and DIO-GFP resulted in similar levels of mCherry and GFP expression (Figure 6A) without spatial interference. Co-infection of the striatum from wild type mice with three different Cre-Off rAAV combinations (DO + DO, DO + FAS, and FAS + FAS) also resulted in expression patterns lacking spatial interference (Figures 6B–D).

## Discussion

### Achieving intermingled Cre-On and Cre-Off rAAV expression

We designed rAAV expression vectors that allow different transgenes to be simultaneously targeted to spatially intermingled cell populations with and without Cre. Although the FLEx system is widely used to achieve Cre-On (DIO) expression, Cre-Off rAAVs are not readily available. Therefore, we used the FLEx system to generate Cre-Off rAAVs (DO) and confirmed the efficacy of this design using striatal injections. High efficiency Cre-On and Cre-Off expression when each system is used in isolation was confirmed both for constitutive genomic Cre expression as well as for acute virally-induced Cre expression.

Whereas DIO and DO rAAVs achieve the expected Cre-On and Cre-Off expression when injected individually, when co-infected, DIO expression was absent in areas with DO expression. This data suggests that many cells within the infection volume were transduced by both DIO and DO rAAV particles, yet expression failed to occur only in Cre expressing cells. However, the presence of Cre and multiple FLEx rAAVs is not sufficient for interference, since DIO/DIO rAAV co-infection led to robust expression from both vectors. The absence of DO expression at the periphery of the example infection was not present in all DIO and DO combinations and thus likely represents a lack of DO rAAV spread and infection rather than Cre-independent interference. Therefore the interference we describe requires Cre and FLEx rAAVs with oppositely oriented transgenes.

Although the exact nature of the destructive, Cre-dependent interference occurring between DIO and DO rAAVs present in the same cell is unknown, we hypothesize that it resulted from intermolecular recombination involving loxP and lox2272 sites. Intermolecular recombination between rAAVs is well documented (Yang et al., 1999). rAAV genomes are single stranded but most undergo second strand synthesis in the nucleus before becoming competent for transgene expression. Double strand synthesis is initiated through self-priming DNA hairpins formed by the inverted terminal repeats (ITRs). These single stranded rAAVs are subject to other types of folding based on DNA complementarity and these interactions can mediate both intra- and inter-molecular recombination (Yang et al., 1999; McCarty et al., 2003) and can be exploited to fuse two independent rAAV genomes into a single expression system (Duan et al., 2000; Nakai et al., 2000; Sun et al., 2000). Lox sites could therefore be a feasible substrate for Cre-dependent intermolecular interactions in the nucleus. Alternatively, inter-molecular recombination could result in the transcription of double-stranded RNAs that alter protein translation by many mechanisms (Blum et al., 2002; Sledz and Williams, 2004; Alvarez et al., 2006).

We describe two solutions for simultaneous Cre-On and Cre-Off expression that are consistent with this idea. First, Cre-Switch rAAVs avoid recombination across heterogenous rAAV vectors since only a single rAAV type is present. Second, rAAVs which use the loxFAS site to achieve Cre-Off expression avoid recombination with DIO rAAVs, since the loxFAS site does not efficiently recombine with loxP or lox2272.

### rAAV and somatic genome interactions involving loxP

Fluorescent Cre-reporter alleles are routinely used to visualize cells that express Cre. The Ai9 reporter uses a loxP flanked stop cassette to prevent TdTomato transcription driven by a CAG promoter (Madisen et al., 2009). Cre-mediated recombination excises the stop cassette allowing TdTomato transcription. This process leaves behind a single loxP site. In mice in which this reporter is used to visualize cells expressing Cre under control of the parvalbumin regulatory sequence, cortical injections of DO-GFP rAAV produced a striking drop in TdTomato fluorescence in the infection area. Similar effects have been observed with DIO rAAVs and in other cell types throughout the brain (Arpiar Saunders, unpublished observation). Thus, FLEx rAAVs should be used with caution in animals with loxP-dependent conditional alleles and the status of the genomic allele must be examined in infected tissue. In contrast, injections with FAS-GFP do not affect reporter fluorescence, indicating that reduced reporter fluorescence with FLEx viruses is not due to rAAV infection or over-expression, but is due to elements, such as the loxP sites, that differ between the FAS and DO/DIO viral genomes.

One intriguing possibility is that the single loxP sites remaining in each of the rAAV and somatic genomes following Cre mediated recombination may mediate integration of rAAV DNA between the CAG promoter and TdTomato coding sequence. Integration of even a single DO rAAV genome (~4.5 kb) might be sufficient to interfere with transcription and be considered, in effect, insertion of an exogenous rAAV-based stop cassette. Since loxFAS sites do not efficiently recombine with loxP, this putative integration would be inefficient in FAS rAAVs (Siegel et al., 2001).

AAV integration into somatic genomes is well documented (Smith, 2008) and Cre-lox can mediate integration reversibly with excision (Tronche et al., 2002). The robust drop in reporter fluorescence suggests a highly efficient and irreversible process, but how DO-GFP integration could become permanent is unclear. Regardless of the mechanism, FLEx rAAVs may interact cryptically with other genomic loxP sites, such as those left behind by conditionally deleted alleles. Once understood, these interactions may be useful for introducing DNA back into targeted genomic sites using somatic loxP and rAAVs.

### Conflict of interest statement

The authors declare that the research was conducted in the absence of any commercial or financial relationships that could be constructed as a potential conflict of interest.
